# Supplementary material for: Single-cell RNA sequencing reveals evolution of immune landscape during glioblastoma progression
Source: Nat Immunol. 2022 May 27;23(6):971–84. doi: 10.1038/s41590-022-01215-0 (PMC9174057; doi:10.1038/s41590-022-01215-0)
Supplement: Supplementary file 1 — Reporting Summary [file 41590_2022_1215_MOESM1_ESM.pdf]

nature research

Corresponding author(s): Vassiliki Boussiotis
Al Charest

Last updated by author(s): Apr 6, 2022

Reporting Summary

Nature Research wishes to improve the reproducibility of the work that we publish. This form provides structure for consistency and transparency in reporting. For further information on Nature Research policies, see our Editorial Policies and the Editorial Policy Checklist.

Statistics

For all statistical analyses, confirm that the following items are present in the figure legend, table legend, main text, or Methods section.

n/a	Confirmed	
☐	☒	The exact sample size (*n*) for each experimental group/condition, given as a discrete number and unit of measurement
☐	☒	A statement on whether measurements were taken from distinct samples or whether the same sample was measured repeatedly
☐	☒	The statistical test(s) used AND whether they are one- or two-sided<br>*Only common tests should be described solely by name; describe more complex techniques in the Methods section.*
☒	☐	A description of all covariates tested
☒	☐	A description of any assumptions or corrections, such as tests of normality and adjustment for multiple comparisons
☐	☒	A full description of the statistical parameters including central tendency (e.g. means) or other basic estimates (e.g. regression coefficient) AND variation (e.g. standard deviation) or associated estimates of uncertainty (e.g. confidence intervals)
☐	☒	For null hypothesis testing, the test statistic (e.g. *F*, *t*, *r*) with confidence intervals, effect sizes, degrees of freedom and *P* value noted<br>*Give P values as exact values whenever suitable.*
☒	☐	For Bayesian analysis, information on the choice of priors and Markov chain Monte Carlo settings
☒	☐	For hierarchical and complex designs, identification of the appropriate level for tests and full reporting of outcomes
☒	☐	Estimates of effect sizes (e.g. Cohen's *d*, Pearson's *r*), indicating how they were calculated

Our web collection on statistics for biologists contains articles on many of the points above.

Software and code

Policy information about availability of computer code

Data collection	Flow Cytometry: Beckman Coulter Gallios with acquisition software Kaluza (v1.1.3), BD LSR Fortessa with acquisition software FACSDiva (8.1), GraphPad Prism (GraphPad Software v9.3.0)<br>R (v3.6.3)
Data analysis	Sequencing Analysis<br><br>Flow Cytometry and<br><br>Patient Data Analysis: Flow cytometry quantification was performing using FLowJo v10.5.3

For manuscripts utilizing custom algorithms or software that are central to the research but not yet described in published literature, software must be made available to editors and reviewers. We strongly encourage code deposition in a community repository (e.g. GitHub). See the Nature Research guidelines for submitting code & software for further information.

Data

Policy information about availability of data

All manuscripts must include a data availability statement. This statement should provide the following information, where applicable:

- Accession codes, unique identifiers, or web links for publicly available datasets
- A list of figures that have associated raw data
- A description of any restrictions on data availability

All data and materials used in the analysis are available in some form to any researcher for purposes of reproducing or extending the analysis. In rare instances, a

material transfer agreement (MTA) may be required. scRNA seq and bulk RNAseq data files are publicly accessible in the Gene Expression Omnibus under accession numbers GSE195848, GSE196174, GSE196175, GSE195813. All analyses and visualizations were performed in R (v3.6.3). Source data are provided with this paper.

Field-specific reporting

Please select the one below that is the best fit for your research. If you are not sure, read the appropriate sections before making your selection.

☒ Life sciences ☐ Behavioural & social sciences ☐ Ecological, evolutionary & environmental sciences

For a reference copy of the document with all sections, see nature.com/documents/nr-reporting-summary-flat.pdf

Life sciences study design

All studies must disclose on these points even when the disclosure is negative.

Sample size	For mouse studies: scRNA seq experiments, no statistical methods were used to predetermine the sample size.<br>For all other mouse studies, a minimum of 3 biological replicates are included in each study. For survival studies, a minimum number of 4 mice are included in each arm. No statistical methods were used to predetermine sample sizes, and our sample sizes were similar to those reported in previous publications<br><br>For patient studies: no statistical methods were used to predetermine the sample size. As many patients as possible were included in the study.
Data exclusions	no data were excluded
Replication	The results from our scRNA-seq experiments were validated by flow cytometry of independent separate cohorts of mice. All of our experiments were performed with biologically independent replicates and were successful at replicating findings.
Randomization	Mice were randomly enrolled in treatment cohorts.
Blinding	scRNA seq samples were blinded to core facility technicians during sample processing. Individuals analyzing data for Fig 2b, c were blinded to the identity of the images analyzed. For all other experiments blinding was not possible because of the nature of the experimental set up.

Reporting for specific materials, systems and methods

We require information from authors about some types of materials, experimental systems and methods used in many studies. Here, indicate whether each material, system or method listed is relevant to your study. If you are not sure if a list item applies to your research, read the appropriate section before selecting a response.

Materials & experimental systems

n/a	Involved in the study	
☐	☒	Antibodies
☐	☒	Eukaryotic cell lines
☒	☐	Palaeontology and archaeology
☐	☒	Animals and other organisms
☐	☒	Human research participants
☒	☐	Clinical data
☒	☐	Dual use research of concern

Methods

n/a	Involved in the study	
☒	☐	ChIP-seq
☐	☒	Flow cytometry
☒	☐	MRI-based neuroimaging

Antibodies

Antibodies used

Flow cytometry antibodies used:
Anti Mouse

Purified CD16/32 BioLegend 101302
Lineage Cocktail FITC BioLegend 133301
Ly-6A/E (Sca-1) PE-Fluor610 ThermoFisher Scientific 61-5981-82
CD127 (IL-7R?) Brilliant Violet 605 BioLegend 135041
CD117 (c-Kit) APC BioLegend 105812
CD16/32 PerCP-Cyanine5.5 BioLegend 101323
CD34 Brilliant Violet 421 BioLegend 119321
CD45 APC/Cyanine7 BioLegend 103116
CD11b PE/Cyanine7 BioLegend 101216
F4/80 Brilliant Violet 605 BioLegend 123133
Ly-6G Brilliant Violet 421 BioLegend 127628
Ly-6C FITC BioLegend 128006

I-A/I-E Alexa Fluor700 BioLegend 107622
CD11c Brilliant Violet 510 BioLegend 117353
CD3 FITC BioLegend 100204
CD279 (PD-1) Brilliant Violet 421 BioLegend 109121
CD152 PE BioLegend 106305
CD278 (ICOS) Brilliant Violet 650 BioLegend 313549
CD274 (BH-H1, PD-L1) PE/Cyanine7 BioLegend 124314
CD366 (Tim-3) PE/Cyanine7 BioLegend 134010
LIVE/DEAD Fixable Aqua Dead Cell Stain Kit ThermoFisher Scientific   L34957
Arg1 APC BioLegend 17-3697-82
CCR7 PE BioLegend 120106
CD11b PE-CF594 BD Biosciences 562287
CD11b PerCP/Cy5.5 BD Biosciences 101228
CD206 PE-cy7 BioLegend 141720
CD3 BV510 BioLegend 100234
CD3 PerCPcy5.5 BioLegend 100218
CD335 PE Cy7 BioLegend 137618
CD4 PE CF594 BD Biosciences 562285
CD44 APC BioLegend 103012
CD45 AF700 BioLegend 103128
CD45 APC R700 BD Biosciences 565478
CD45 APC Cy7 BD Biosciences 557659
CD49d af488 BioLegend 103611
CD62L PE BioLegend 104408
CD8 BB515 BD Biosciences 564527
CD8 APC Cy7 BD Biosciences 557760
CD80 PE Cy7 BioLegend 104734
CD86 af488 BioLegend 105018
CTLA4 PE BioLegend 106306
EdU AF488 Invitrogen C10632
EGFR (AY13) PerCPcy5.5 BioLegend 352914
EGFR af488 BioLegend 352907
FOXP3 BV421 BioLegend 126419
Galectin 1 PE R&D systems IC1245P
Galectin 9 APC BioLegend 137912
Granzyme B PE Cy7 ThermoFisher Scientific 25-8898-82
Ki67 BV421 BioLegend 652411
Lag3 PE Cy7 BioLegend 125226
LY6C APC Cy7 BioLegend 128026
Ly6C APC BioLegend 128016
LY6G PE BioLegend 127608
Ly6G PEcy7 BioLegend 127618
MHCI PerCPcy5.5 BioLegend 107626
MHCII BV421 BioLegend 107632
P2ry12 APC BioLegend 848006
PD-1 PerCP Cy5.5 BioLegend 109120
PDGFRA (APA5) APC BD Biosciences 562777
PDL1 PE BioLegend 155404
PDL2 BV421 BioLegend 564245
Tim3 APC BioLegend 134008
TNF APC Cy7 BioLegend 506344
Viability Zombie Yellow BioLegend 423104
Viability Zombie NIR BioLegend 423106
Vista af488 BioLegend 143720

Anti Human

MRP-14 FITC BioLegend 350703
CD49d APC BioLegend 304308
CD33 PE-CF594 BD Biosciences 562492
CD16 Alexa Fluor700 BioLegend 302025
HLA-DR-PE/Cy7 BioLegend 307615
CD15 PerCP/Cy5.5 BioLegend 323019
CD45 APC-H7 BD Biosciences 560274
CD14-VioBlue Miltenyi Biotec 130-098-058
CD4 PE-CF594 BD Biosciences 562316
CTLA-4 PE eBioscience 12-1529-42
CD45 Alexa Fluor700 Biolegend 368513
TIM3 (CD366) APC Biolegend 345012
FOXP3 PE-Cy7 eBioscience 25-4777-42
Human TruStain FcX Biolegend 422301
CD45 Alexa Fluor700 Biolegend 304023
HLA-DR FITC BD Biosciences 562008
CD8 BB515 BD Biosciences 564526
CD3 BV510 BD Biosciences 563109
CD11b/Mac-1 PE BD Biosciences 561001

PD-1 (CD279) PerCP-Cy5.5 BD Biosciences 561273
LAG-3 (CD223) PE/Cy7 Biolegend 369310
CD14 PE-Vio770 Milentyi Biotech 130-098-074

Validation	All anti mouse antibodies were validated and optimized on mouse tissues and all anti human antibodies were validated and optimized on human tissues by the source companies as they are all commercially available. All antibodies used were evaluated by the manufacturers as provided on their websites.

Eukaryotic cell lines

Policy information about cell lines

Cell line source(s)	for HEK293T, source ATCC catalog CRL3216
Authentication	No authentication was applied as these cells are commercially available.
Mycoplasma contamination	Cells tested negative for mycoplasm using a sensitive commercial PCR assay (Lookout kit, MP0035)
Commonly misidentified lines (See ICLAC register)	n/a

Animals and other organisms

Policy information about studies involving animals; ARRIVE guidelines recommended for reporting animal research

Laboratory animals	mus musculus, conditional transgenic for EGFR wild type, constitutive loss of Cdkn2a, conditional PTEN lox/lox, and conditional Lox-STOP-lox Luciferase on a mixed background. Both males and females 1:1 ratio of >6 weeks of age were used in these studies.
Wild animals	No wild animals were used in this research
Field-collected samples	No field-collected samples were used in this research
Ethics oversight	All mouse procedures were carried out in accordance with Beth Israel Deaconess Medical Center recommendations for care and use of animals and were maintained and handled under protocols approved by Institutional Animal Care and Use Committee (IACUC).

Note that full information on the approval of the study protocol must also be provided in the manuscript.

Human research participants

Policy information about studies involving human research participants

Population characteristics	Human samples were obtained from glioma patients undergoing debulking resection surgery. 7 males and 6 females, ages ranging from 29 to 70 years old.
Recruitment	The human samples used in this study are human subject and protected under IRBs from BIDMC and MGH. Obtained informed consent were obtained under those IRBs. Human samples were de identified. Patients were recruited on basis of their survival surgical needs. There are no self selection bias or any other bias from recruitment as these surgical procedures are life saving and do not impact research.
Ethics oversight	BIDMC IRB and MGH IRB.

Note that full information on the approval of the study protocol must also be provided in the manuscript.

Flow Cytometry

Plots

Confirm that:

- [x] The axis labels state the marker and fluorochrome used (e.g. CD4-FITC).
- [x] The axis scales are clearly visible. Include numbers along axes only for bottom left plot of group (a 'group' is an analysis of identical markers).
- [x] All plots are contour plots with outliers or pseudocolor plots.
- [x] A numerical value for number of cells or percentage (with statistics) is provided.

Methodology

Sample preparation	Cells from tumor tissues were isolated as described in the Methods section. After isolation, cells were analyzed for cell surface or intracellular markers using fluorophore-conjugated antibodies. Antibody staining was performed in 1X PBS and

intracellular staining was performed using the eBioscience FoxP3/TF kit. Cells were stained for 30 min in dark then analyzed by flow cytometry as described in Methods. Compensation was performed using fluorescence minus one.

Instrument	Beckman Coulter Gallios<br>BD LSR Fortessa
Software	Acquisition software Kaluza (Beckman Coulter Gallios)<br>Acquisition software FACSDiva (BD LSR Fortessa)<br>Quantification software FlowJo v10.5.3
Cell population abundance	For scRNAseq CD45+ and CD45- populations were flow sorted and average viability was 66% and 87% respectively. Percent of CD45+ cells ranged between 6.8% to 44% with percent of CD45- 56% to 93.2%.
Gating strategy	Cells were first gated based on size using Forward and Side scatter, followed by identification of singlets using FSC-H and FSC-A.<br>To isolate cells for the single cell experiments, freshly dissociated GBM tumor cells were gated as follows for the sorted populations: Non-immune cells (CD45-), Immune cells (CD45+).<br><br>For validation and other flow cytometry experiments, freshly dissociated GBM tumor cells were gated as follows for these populations of cells:<br>EGFR positive cancer cells (CD45-EGFR+),<br>Macrophages CD45highCD11b+Ly6C-Ly6G-P2ry12-<br>Microglia CD45lowCD11b+Ly6C-Ly6G-P2ry12+<br>PMN-MDSCs CD45+CD11b+Ly6c+Ly6G+<br>M-MDSCs CD45+CD11b+Ly6c+Ly6G-<br>CD8+ T cells CD45+CD3+CD8+CD4-<br>CD4+ T cells CD45+CD3+CD4+CD8-<br>Regulatory T cells CD45+CD3+CD4+CD8-Foxp3+<br><br>Mouse bone marrow cells were gated for the following populations:<br>GMP: Lin-, Sca1-, CD127-, c-kit+, CD16/32+<br>CMP: Lin-, Sca1-, CD127-, c-kit+, CD16/32-<br>LK: Lin-, Sca1-, CD127-, c-kit+<br>LSK: Lin-, Sca1+, CD127-, c-kit+<br><br>Mouse spleen cells were gated as follows:<br>myeloid cells CD45+CD11b+,<br>T cells CD45+CD3+<br><br>For human glioma freshly dissociated tumor cells:<br>PMN-MDSC, neutrophil, granulocyte CD45+; CD11b+; Ly6G+; Ly6C+<br>M-MDSC, monocyte CD45+; CD11b+; Ly6G-; Ly6C+<br>Tumor associated Macrophages CD45+; CD11b+; Ly6G-; Ly6C-<br>CD4 T-cell CD45+; CD3+; CD4+<br>CD8 T-cell CD45+; CD3+; CD8+<br>CD8 Effector memory T-cell (TEM) CD45+; CD3+; CD8+; CD44+; CD62L-<br>CD8 Central memory T-cell (TCM) CD45+; CD3+; CD8+; CD44+; CD62L+<br>CD8 Naïve T-cell CD45+; CD3+; CD8+; CD44-; CD62L+<br>CD4 Effector memory T-cell (TEM) CD45+; CD3+; CD4+; CD44+; CD62L-<br>CD4 Central memory T-cell (TCM) CD45+; CD3+; CD4+; CD44+; CD62L+<br>CD4 Naïve T-cell CD45+; CD3+; CD4+; CD44-; CD62L+<br>Regulatory T-cell CD45+; CD3+; CD4+; Foxp3+

☒ Tick this box to confirm that a figure exemplifying the gating strategy is provided in the Supplementary Information.
